# Shared signatures between rheumatoid arthritis, systemic lupus erythematosus and Sjögren’s syndrome uncovered through gene expression meta-analysis

**DOI:** 10.1186/s13075-014-0489-x

**Published:** 2014-12-03

**Authors:** Daniel Toro-Domínguez, Pedro Carmona-Sáez, Marta E Alarcón-Riquelme

**Affiliations:** Area of Medical Genomics, Pfizer–Universidad de Granada–Junta de Andalucía de Genómica e Investigación Oncológica (GENyO), Parque Tenológico de la Salud Fundación (PTS) Granada, Avenida de la Ilustración, 114-18016 Granada, Spain; Bioinformatics Unit, Pfize-Universidad de Granada-Junta de Andalucia. Centro de Genómica e investigación Oncológica (GENyO), Parque Tenológico de la Salud Fundación (PTS) Granada, Avenida de la Ilustración, 114-18016 Granada, Spain; Arthritis and Clinical Immunology, Oklahoma Medical Research Foundation, 825 NE 13th Street, Oklahoma City, OK 73104 USA

## Abstract

**Introduction:**

Systemic lupus erythematosus (SLE), rheumatoid arthritis (RA) and Sjögren’s syndrome (SjS) are inflammatory systemic autoimmune diseases (SADs) that share several clinical and pathological features. The shared biological mechanisms are not yet fully characterized. The objective of this study was to perform a meta-analysis using publicly available gene expression data about the three diseases to identify shared gene expression signatures and overlapping biological processes.

**Methods:**

Previously reported gene expression datasets were selected and downloaded from the Gene Expression Omnibus database. Normalization and initial preprocessing were performed using the statistical programming language R and random effects model–based meta-analysis was carried out using INMEX software. Functional analysis of over- and underexpressed genes was done using the GeneCodis tool.

**Results:**

The gene expression meta-analysis revealed a SAD signature composed of 371 differentially expressed genes in patients and healthy controls, 187 of which were underexpressed and 184 overexpressed. Many of these genes have previously been reported as significant biomarkers for individual diseases, but others provide new clues to the shared pathological state. Functional analysis showed that overexpressed genes were involved mainly in immune and inflammatory responses, mitotic cell cycles, cytokine-mediated signaling pathways, apoptotic processes, type I interferon–mediated signaling pathways and responses to viruses. Underexpressed genes were involved primarily in inhibition of protein synthesis.

**Conclusions:**

We define a common gene expression signature for SLE, RA and SjS. The analysis of this signature revealed relevant biological processes that may play important roles in the shared development of these pathologies.

**Electronic supplementary material:**

The online version of this article (doi:10.1186/s13075-014-0489-x) contains supplementary material, which is available to authorized users.

## Introduction

*Autoimmunity* refers to the failure of the immune system to recognize its own constituent parts, eliciting an immune response against the tissues themselves. Currently, there are more than 80 clinically distinct autoimmune diseases [1], and the biological mechanisms that cause them are not clearly understood. It has been suggested that both genetic and environmental factors influence the development of autoimmune diseases [2]. Three of these inflammatory, autoimmune diseases are systemic lupus erythematosus (SLE), rheumatoid arthritis (RA) and Sjögren’s syndrome (SjS).

Although at first appearance these disorders have different phenotypes, they all are heterogeneous, multifactorial disorders that share molecular mechanisms which elicit similar clinical and pathogenic features. In fact, a differential diagnosis between these immune disorders at an early stage is not always reliable, and treatments are similar for all three, except when organ damage ensues or features of one dominate over another. Therefore, one of the major aims in this field is to discover similarities and differences at the molecular level between these diseases and between groups of patients across diseases. This will lead to a better understanding of the specific biological mechanisms and the development of more efficient and personalized treatments.

In this context, the analysis of gene expression patterns can provide useful information for understanding the molecular mechanisms by defining specific gene expression signatures that underlie these disorders. These studies are becoming more plentiful as a result of the development of high-throughput technologies such as microarrays and next-generation sequencing. These methods allow us to measure gene expression on a genome-wide scale, including for autoimmune diseases, and have been widely used during the past decade (see, for example, [3-5]). In this field, meta-analysis techniques offer the potential to integrate and jointly analyze data from different sources. In previous meta-analyses, investigators have combined data related to the same disease from different studies to get more consistent and reliable results. For example, Song *et al*. [6] performed a meta-analysis integrating three public SjS datasets. Arasappan *et al*. [7] conducted a pathway-based meta-analysis of four SLE datasets and identified a 37-gene signature associated with this disease. Olsen *et al*. [8,9] analyzed different RA datasets and found several genes related to pathways such as type I interferon (IFN), apoptotic processes and cell cycles.

Moreover, meta-analytic techniques have also been used to integrate data from different diseases to uncover similar patterns. In this context, Tuller *et al*. [4] analyzed public data on six different autoimmune diseases (multiple sclerosis, SLE, juvenile RA, Crohn’s disease, ulcerative colitis and type 1 diabetes) from peripheral blood mononuclear cells (PBMCs). Silva *et al*. [10] combined SLE and RA data to uncover coexpression patterns, and Higgs *et al*. [11,12] integrated data on SLE, myositis, RA and scleroderma and defined a common type I IFN-related signature.

In this study, we performed a gene expression meta-analysis using publicly available gene expression data from PBMC samples of SLE, RA and SjS patients and controls. To the best of our knowledge, this is the first study in which gene expression data from these three diseases have been integrated, together with analysis of the common gene expression signatures with respect to healthy controls.

## Methods

### Search and selection of datasets

We mined the National Center for Biotechnology Information (NCBI) Gene Expression Omnibus (GEO) database [13] to find all publicly available gene expression datasets related to SLE, RA and SjS. From among all published studies, we selected for our analysis those that fit the following criteria: (1) They had to include control and case samples in separate arrays (one-channel arrays); (2) they had to have been performed with human PBMC samples; and (3) the samples had to have been obtained without any type of treatment.

### Processing of the datasets and meta-analysis

Initial processing of the data was carried out using the R statistical programming language. Each dataset was downloaded from the NCBI GEO database using the GEOquery R package [14], and probes were annotated with the Entrez Gene identifiers, which were used to merge data from different platforms for further analysis. In each dataset, gene expression profiles were averaged for duplicate genes by computing the median values. Genes with missing values in more than 10% of samples were filtered out, and the remaining missing values were imputed using the average expression values within the group (case or control). The integration of different datasets and gene expression meta-analysis was performed using the INMEX software package [15]. Gene expression values were log-transformed and normalized by applying quantile normalization. The dataset for identification of genes specifically overexpressed in lupus CD4 T and B cells [GEO:GSE4588] contains samples from SLE and RA patients; therefore, these two subpopulations were treated as two different datasets.

Differential expression meta-analysis across diseases and healthy controls was carried out by using a random effects model (REM) [16,17], which is based on combining the effect sizes (ESs) or changes of gene expression from different studies and obtaining an overall mean.

### Functional analysis

In order to obtain biological information from the list of differentially expressed genes, we performed a functional analysis using the GeneCodis tool [18-21]. This software allows evaluation of which annotations are significantly enriched in a gene list, which can be used as functional descriptors of the biological processes that are acting in experimental conditions [19,20]. Gene Ontology (GO) and Kyoto Encyclopedia of Genes and Genomes pathway annotations were evaluated in the analysis.

## Results

### Studies selected for the meta-analysis

After conducting a thorough search, we identified 19 datasets related to SLE, RA and SjS. From among this initial set, we dismissed three datasets that included treated samples, four datasets without control samples, five with too many missing values (more than 50% of missing values) and three generated with two-channel arrays. Thus, our final meta-analysis included four datasets (Table 1). The selected datasets comprise a total of 371 samples with a breakdown of 94 controls, 190 SjS samples, 54 SLE samples and 33 RA samples.

**Table 1:** **Datasets used in the study** ^**a**^

**GEO ID**	**Platform**	**Disease**	**Cases/controls**	**Description**	**Reference**	**Key findings**
[GEO:GSE10325]	Affymetrix Human Genome U133A Array	SLE	39/28	Expression data from human peripheral blood subsets	[22]	Some apoptotic genes are related to SLE phenotype
[GEO:GSE15573]	Affymetrix Human Genome U133 Plus 2.0 Array	RA	18/15	Immunity and defense genes in PBMCs of RA patients	[23]	283 underexpressed genes (involved in metabolic processes), 101 overexpressed (immunity, calcium transport)
[GEO:GSE4588]	Illumina Human 6 v2.0 expression BeadChip	SLE, RA	15 SLE, 15 RA/19	Identification of genes specifically overexpressed in SLE CD4 T and B cells	–	–
[GEO:GSE51092]	Illumina Human WG-6 v3.0 expression BeadChip	SjS	190/32	Variants at multiple loci implicated in both innate and adaptive immune responses are associated with SjS	[24]	Risk loci for SjS (related to IFN pathway, STATs, chemokine receptors, interleukins proteins or a BLK

^a^NCBI GEO ID: Unique gene expression series identifier for each dataset in the National Center for Biotechnology Information Gene Expression Omnibus database; Platform: Microarray platform; Disease: Type of disease and number of cases/controls in each dataset; Description: Brief description of the study; Reference: Publication; Key findings: Main findings in the original studies. BLK, B Lymphocyte Kinase Protein; IFN, Interferon; PBMCs, Peripheral blood mononuclear cells; RA, Rheumatoid arthritis; SLE, Systemic lupus erythematosus; SjS, Sjögren’s syndrome; STAT, Signal transducers and activators of transcription.

### Identifying a common gene expression signature among systemic lupus erythematosus, rheumatoid arthritis and Sjögren’s syndrome

For meta-analysis, the processed data were loaded into the INMEX web tool, and ES statistical analysis was used to find genes that were differentially expressed among diseases and healthy controls across different studies. Rather than requiring us to merge the original datasets, this method allowed us to combine them through high-level summary statistics, thus avoiding the problem of interstudy variation.

We identified 412 genes that were consistently differentially expressed (*P* < 0.05). Among the different studies (see Additional file 1), 210 genes were overexpressed and 202 were underexpressed.

**Figure 1:**
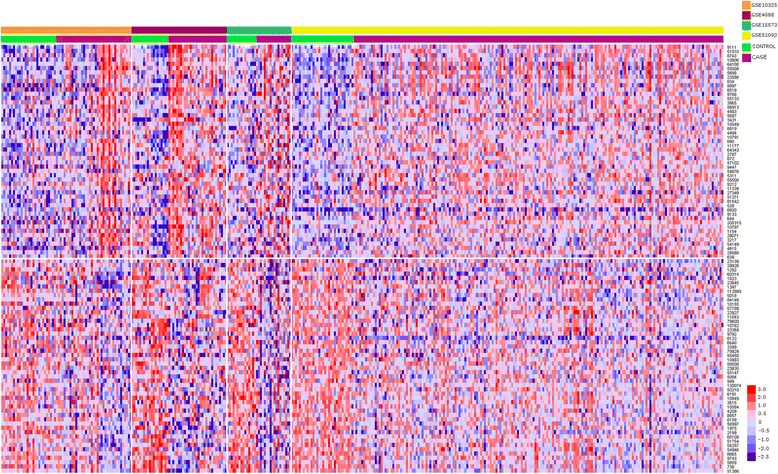
**Heatmap of top differentially expressed genes.** The heatmap represents the log_2_- transformed expression values with the top 50 overexpressed (top) and top 50 underexpressed (below) genes.

Among this initial set, we found 187 overexpressed genes in all diseases with respect to healthy controls and 184 that were underexpressed. Additional file 1 gives the average fold changes of each gene in all datasets. This set comprises the common gene expression signature—that is, genes that are significantly differentially expressed in all diseases with respect to healthy controls. Figure 1 shows the top 50 over- and underexpressed genes.

Some of the most differentially expressed genes were *HERC6*, which belongs to the HERC family of E3 ubiquitin ligases, and *RTP4*, which encodes a receptor (chemosensory) transporter protein related to “chemotaxis” and was previously described as an IFN-inducible gene [25]. In addition, the most overexpressed genes with the largest ESs were *RSAD2* and *IFI44L. IFI44L* encodes the IFN-induced protein 44-like, which has been described in several autoimmune diseases in conjunction with other genes involved in the type I IFN signaling pathway, such as *IFIT1*, *IFI27* and *IFITM1*. We also found overexpression of these genes in our results.

The gene with the lowest ES (ES = −1.2545) was eukaryotic translation elongation factor 2 (*EEF2*), a biomarker protein of some types of cancer [26] that plays an important role in protein synthesis. This was, in fact, the most relevant pathway that was associated with underexpressed genes.

Meta-analytic techniques have been also used to evaluate reproducibility and bias across microarray studies. This is especially important when comparing replicated samples or samples of the same condition or phenotype. In this sense, there are different methods that can be used for this purpose [27]. In this context, we also evaluated the meta-analysis results with those obtained from individual analyses of studies and/or diseases. We found 132 gained genes and 2,168 lost genes in our meta-analysis (see Additional file 1). Gained genes are the differentially expressed genes identified only in the meta-analysis and not in the individual analysis, because they show weak signals but consistent expression patterns across the different datasets. Lost genes are genes identified as differentially expressed genes in any individual analysis, but not in the meta-analysis. These genes show either conflicting changes in expression profiles or very large variations across different studies [6,15]. Additional file 2 contains a detailed study of the different datasets and the analysis used in our study.

### Functional and pathway analysis

**Figure 2:**
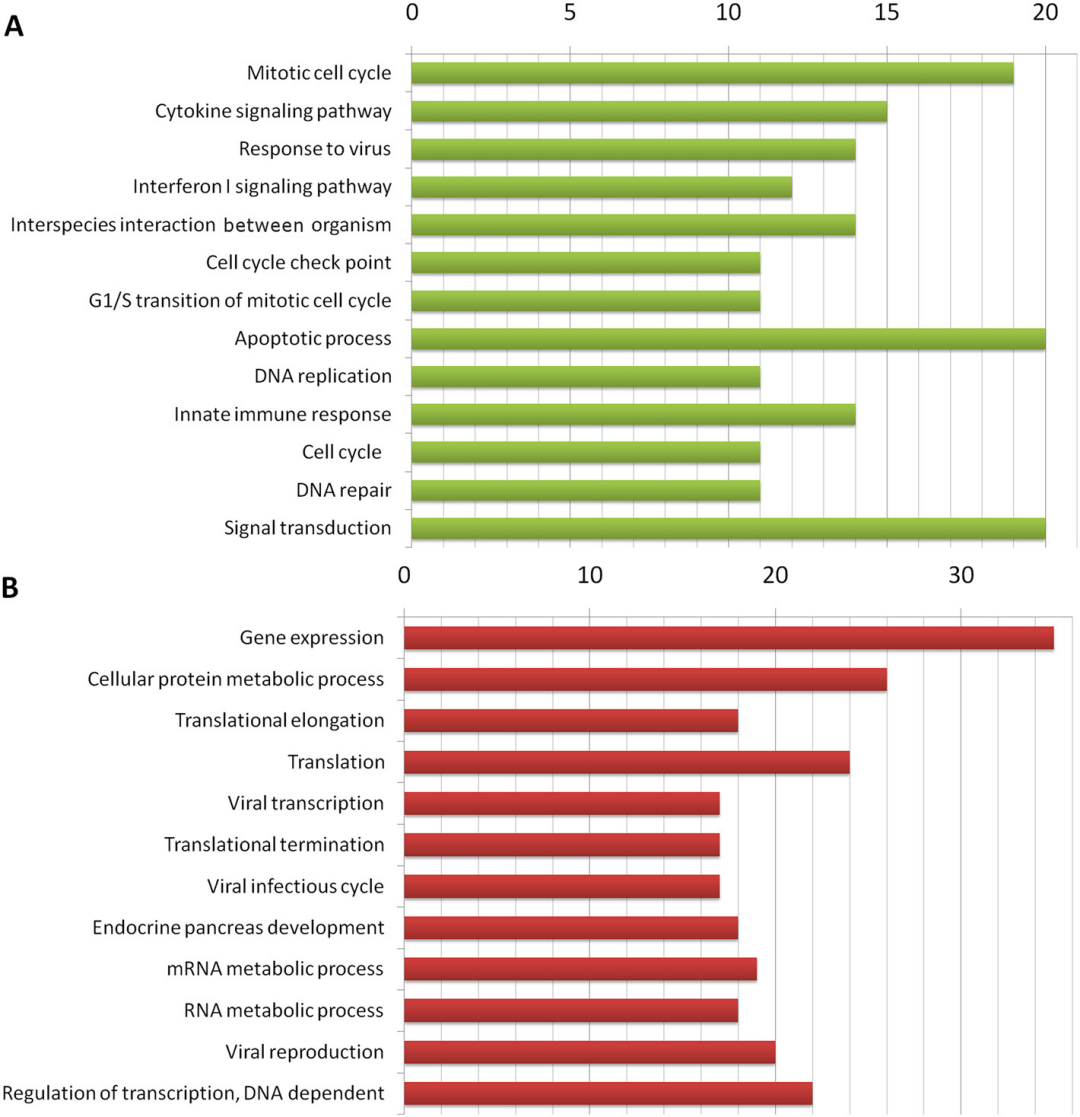
**Biological function related to the differentially expressed genes.** This graphic shows the main Gene Ontology (GO) biological functions identified and related to overexpressed genes **(A)** and underexpressed genes **(B)**. GO annotations were considered significantly enriched in the list of genes if they had a *P*-value <0.01 and were associated with at least ten genes. The *x*-axis represents the number of genes, and the *y*-axis shows the names of the significant GO categories sorted by decreasing *P*-values.

For the analysis of biological processes associated with the differentially expressed genes, we evaluated the enrichment of functional annotations using the GeneCodis tool [18]. GO annotations for biological processes were significantly overrepresented in the gene list if they showed a *P*-value <0.05. Results for biological pathways of overexpressed and underexpressed genes are shown in Figure 2 and Additional file 1. Functions such as “mitotic cell cycle,” “cytokine-mediated signaling pathway,” “response to virus” or “type I IFN-mediated signaling pathway” or “immune response” were significantly associated with this set of genes. This is in agreement with previous work that has associated these pathways with each of the diseases [12,28-31].

Similarly, the most significant GO categories or pathways in the analysis of underexpressed genes were “gene expression” and others related to protein biosynthesis mechanisms previously reported [32].

## Discussion

In this study, we define a signature of differentially expressed genes for SLE, RA and SjS using a gene expression meta-analytic strategy showing common biological mechanisms across three otherwise clinically separate entities. The combined ES and REM meta-analytic method was chosen because it allowed us to integrate microarray datasets from different platforms consistently, without the obstacle of the batch effects that we clearly observed when we began our analyses. We performed the meta-analysis using four publicly available datasets and defined a common signature composed of 187 overexpressed genes and 184 underexpressed genes in all diseases compared to healthy controls. We found significant pathways related to overexpressed genes, such as “immune response,” “type I IFN-mediated signaling pathway,” “cytokine-mediated signaling pathway,” “mitotic cycle” and “response to virus,” as well as pathways related to underexpressed genes that highlight gene expression and metabolic processes.

In this context, independent studies of SLE, RA and/or SjS have shown that overexpression of type I IFN-related genes is very consistent [28-31,33,34], and it appears that these genes roughly form a common pattern in all inflammatory autoimmune diseases [4,6,7,9-12]. The cytokine signaling pathway-related genes behave similarly. In fact, both routes are tightly related. IFN proteins regulate several signaling pathways normally in response to pathogens, such as apoptosis or immune stimulation. In the gene expression signature, we identified several type I IFN-related genes as the most significantly overexpressed genes in all diseases, such as *IFI44L*, *IFI44*, *IFI27* and *IFIT1*. We also found other genes, such as *JAK2*, involved in the Janus kinase/signal transducer and activator of transcription (JAK/STAT) signaling pathway and previously related to immune disorders [35]. JAK/STAT signaling promotes IFN-stimulated gene transcription. *OASL* is and IFN-inducible protein related to antiviral activity [36]. In addition, we found genes related to apoptosis, with genes such as *FAS*, which has been described as a risk allele in some autoimmune diseases [37,38]; *TNFSF10*; and *CASP1*. We also identified several proteasome subunits, such as PSM2, PSM6 and PSMC2. Apoptotic processes have been related to autoimmune diseases in different studies [39]. Apoptotic cells are not immunologically neutral, and the accumulation of apoptotic material not properly phagocytosed can be an important source of autoimmune antigens, enabling the development of autoimmune disorders [40]. In this context, there are some hypotheses focused on the increase in apoptosis, lazy phagocytes or the interaction between apoptotic material and antigen-presenting cells as potential triggers for these diseases.

In addition, some of these genes have been described previously as biomarkers of one or a variety of autoimmune diseases, such as IFN-induced protein 44-like, chemokine receptor 1 and FAS. Therefore, our results are consistent with previously published data for each of the three disorders, but show, for the first time to our knowledge, and formally, their shared genetic signatures.

Moreover, we found interesting results, such as the overexpression of *EIF2AK2* gene (or PKR) (see Additional file 1). This gene is initially related to the response to virus and the innate immune response and encodes a serine/threonine protein kinase that is activated by autophosphorylation after binding to double-stranded RNA [41]. The activated form can phosphorylate multiple substrates, including several translation initiation factors, such as eIF2A, eIF3F eIF2S1 and eEF2, impairing the recycling of these factors between successive rounds of initiation and leading to inhibition of translation, which eventually results in shutdown of cellular protein synthesis and a reduction in cell proliferation [42,43]. This is in agreement with our finding of biological processes related to inhibition of gene expression and protein synthesis in the list of underexpressed genes.

In a previous study of SLE, Groulleau *et al*. [32] described the relationship between PKR and the phosphorylation of the eIF2A translation initiation factor, but this action was attributed only to SLE, whereas researchers in other studies independently related PKR with RA [44,45]. In addition, PKR phosphorylates p38, JNK and nuclear factor κB (NF-κB), which are proteins of the mitogen-activated protein kinase signaling pathway related to the production of cytokines and tumor necrosis factor [46]. These in turn intervene in apoptotic processes, regulation of signal transduction or cell proliferation and differentiation. PKR has direct influences on the production of IFNs [47,48]. We also found other genes related to the immune system, such as *MYD88*, which is an adaptor protein of Toll-like receptors that activates NF-κB and translocates to the nucleus to stimulate the expression of certain genes for the production of cytokines and IFN proteins [49].

Regarding the pathways related to underexpressed genes, the two most relevant processes were “gene expression” and “cellular protein metabolic process”. Analysis of genes associated with these annotations revealed that many were genes involved in translation, such as ribosomal protein-encoding genes, and different eukaryotic translation initiation factor subunits, such as eEF2 or eIF3F mentioned above. The relationship between the underexpression of these genes and autoimmune disease is largely undefined. We also found genes involved in translation and cell growth, which are underexpressed, as mentioned above.

## Conclusions

We performed a gene expression meta-analysis using previously published datasets obtained from PBMCs of SLE, RA and SjS patients. A common gene expression signature was defined, comprising many genes that have been previously related to one, two or each of the three diseases. Although there are previous gene expression meta-analyses of immune-related diseases, our present study is the first one, to our knowledge, in which data on these three specific disorders have been integrated, which allowed us to define common biological processes. We found that pathways in our results, such as “type I IFN-mediated signaling pathway,” apoptotic processes, “immune response,” reduction in translation processes and “response to virus.” This suggests that a majority of these pathways are related to the action of the IFN proteins. However, we found other pathways, such as mitotic cell cycles, whose relationship to the IFN pathway, or even to the diseases themselves, has not been described. Future functional and specific studies of the genes we identified are needed to define the roles of these genes in the pathogenesis of SADs.

## Additional files

Additional file 1:
**Four Excel spreadsheets with results from the meta-analysis.** **Spreadsheet 1:** Differential gene expression analysis results with list of the names of the genes and their Entrez ID, the value of the combined ESs among the different experiments for each gene and the associated *P*-values. The left column shows the upexpressed genes, and the right column shows the underexpressed genes. **Spreadsheet 2:** The fold changes of each gene in all datasets. Genes marked with an asterisk were removed from the gene expression signature, as they did not show a consistent pattern of overexpression or underexpression in all diseases. **Spreadsheet 3:** Gain and loss gene lists. **Spreadsheet 4:** All GO categories enriched in the gene signature are listed, with the minimum number of genes set to three. The table contains the number of genes in each pathway, the corrected *P*-values (Hyp*), the GO annotation numbers and the names of the pathways and the genes related to each pathway. Additional file 2:
**Detailed study of the different datasets used and their influence in the final results of the meta-analysis.**

## Abbreviations

ES: Effect size
GEO: Gene Expression Omnibus
GO: Gene ontology
IFN: Interferon
JAK/STAT: Janus kinase/signal transducer and activator of transcription
NCBI: National Center for Biotechnology Information
NF-κB: Nuclear factor κB
PBMC: Peripheral blood mononuclear cell
RA: Rheumatoid arthritis
REM: Random effects model
SAD: Inflammatory and systemic autoimmune disease
SjS: Sjögren’s syndrome
SLE: Systemic lupus erythematosus
