# Supplementary material for: Shared signatures between rheumatoid arthritis, systemic lupus erythematosus and Sjögren’s syndrome uncovered through gene expression meta-analysis
Source: Arthritis Res Ther. 2014 Dec 3;16(6):489. doi: 10.1186/s13075-014-0489-x (PMC4295333; doi:10.1186/s13075-014-0489-x)
Supplement: Additional file 2: — Detailed study of the different datasets used and their influence in the final results of the meta-analysis. [file 13075_2014_489_MOESM2_ESM.pdf]

Additional Figures

We compared the results from individual datasets and those obtained in the meta-analysis. Figure S1 shows gained and lost genes. Figure S2 shows the comparison of dataset-based analysis and the meta-analysis while figure S3 shows the results from the different diseases. Finally, Table 3 represents the over-lapping genes among different datasets. It is noteworthy that the dataset GSE51092 (SjS) is the one that share more significant genes with the meta-analysis results and with the results from individual datasets.

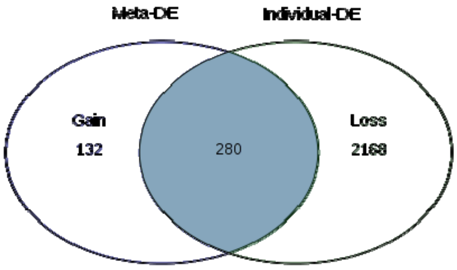

Figure S1. Venn diagram with gain and loss genes

The Venn diagram showing overlap between differentially expressed genes identified from the meta-analysis (Meta-DE) and those from each individual data analysis (individual-DE)

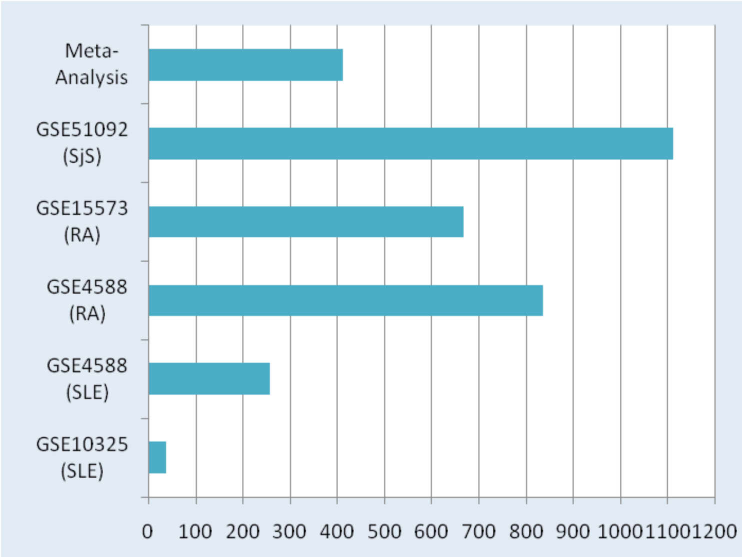

Figure S2. Differentially expressed genes in individual datasets and in the meta-analysis.

The y-axis contains represents the different datasets and x-axis number of genes found differentially expressed. GSE4588 contains samples from lupus and arthritis, therefore,

we created two subsets of this data set belonging to each disease and we treat them as independent datasets. Large differences can be noted among the results from 39 significant genes found in dataset GSE10325 to 1110 significant genes found in dataset GSE51092. Interestingly, dataset some SLE samples from dataset GSE10325 showed a expression pattern similar to controls (see heatmap in the main manuscript). This pattern was also present in the results discussed in the original publication. Removing these samples increased the number of differentially expressed genes found in the meta-analysis (data not shown), which may reflect the heterogeneity of the disease.

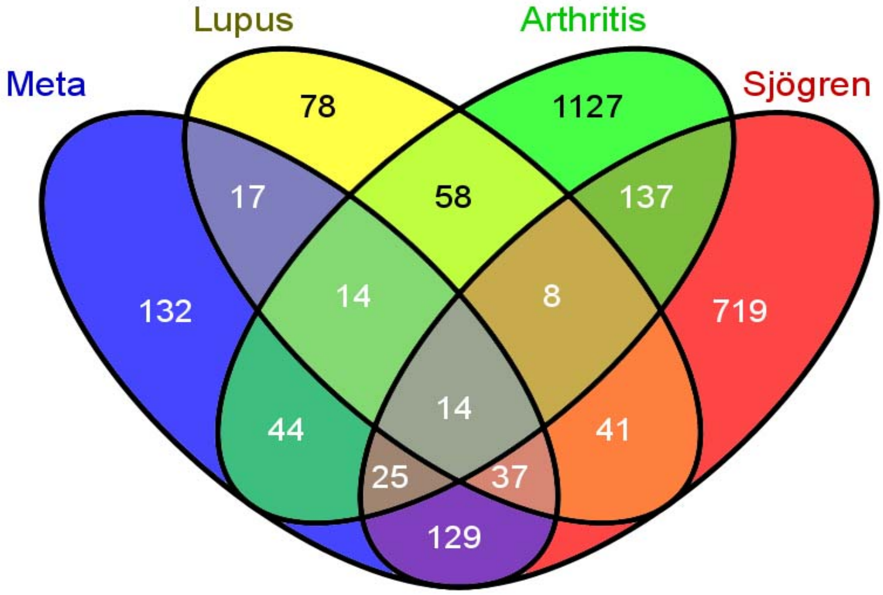

Figure S3. Venn diagram showing over-lapping genes among individual disease based analysis and meta-analysis

This diagram shows the genes overlap among the three diseases and the meta-analysis. We define the set of differentially expressed genes in each disease by merging results from the analysis of different datasets related to the same disease. Genes found from the analysis of SjS samples showed the highest overlap with genes from the meta-analysis, followed by RA and SLE. Obviously this was influenced by the number of genes found from individual analysis.

	Significant genes	Meta-analysis	GSE10325 (SLE)	GSE4588 (SLE)	GSE4588 (RA)	GSE15573 (RA)	GSE51092 (SjS
GSE10325 (SLE)	39	21		29	6	2	29
GSE4588 (SLE)	257	79			73	26	95
GSE4588 (RA)	668	63				77	87
GSE15573 (RA)	836	44					109
GSE51092 (SjS	1110	205					

Table S1. This table represents the number of differentially expressed genes in each individual dataset (column 1), the number of common genes with the meta-analysis results (column 2) and the number of common genes among different datasets (columns 3- to 7).
